# A multiscale mechanistic model of human dendritic cells for *in-silico* investigation of immune responses and novel therapeutics discovery

**DOI:** 10.3389/fimmu.2023.1112985

**Published:** 2023-03-10

**Authors:** Sara Sadat Aghamiri, Bhanwar Lal Puniya, Rada Amin, Tomáš Helikar

**Affiliations:** Department of Biochemistry, University of Nebraska-Lincoln, Lincoln, NE, United States

**Keywords:** systems immunology, predictive modeling, *In-Silico* experiments, antigen-presenting cell, dendritic cell, immunology highlights

## Abstract

Dendritic cells (DCs) are professional antigen-presenting cells (APCs) with the unique ability to mediate inflammatory responses of the immune system. Given the critical role of DCs in shaping immunity, they present an attractive avenue as a therapeutic target to program the immune system and reverse immune disease disorders. To ensure appropriate immune response, DCs utilize intricate and complex molecular and cellular interactions that converge into a seamless phenotype. Computational models open novel frontiers in research by integrating large-scale interaction to interrogate the influence of complex biological behavior across scales. The ability to model large biological networks will likely pave the way to understanding any complex system in more approachable ways. We developed a logical and predictive model of DC function that integrates the heterogeneity of DCs population, APC function, and cell-cell interaction, spanning molecular to population levels. Our logical model consists of 281 components that connect environmental stimuli with various layers of the cell compartments, including the plasma membrane, cytoplasm, and nucleus to represent the dynamic processes within and outside the DC, such as signaling pathways and cell-cell interactions. We also provided three sample use cases to apply the model in the context of studying cell dynamics and disease environments. First, we characterized the DC response to Sars-CoV-2 and influenza co-infection by *in-silico* experiments and analyzed the activity level of 107 molecules that play a role in this co-infection. The second example presents simulations to predict the crosstalk between DCs and T cells in a cancer microenvironment. Finally, for the third example, we used the Kyoto Encyclopedia of Genes and Genomes enrichment analysis against the model’s components to identify 45 diseases and 24 molecular pathways that the DC model can address. This study presents a resource to decode the complex dynamics underlying DC-derived APC communication and provides a platform for researchers to perform *in-silico* experiments on human DC for vaccine design, drug discovery, and immunotherapies.

## Highlights

- The predictive model of the human Dendritic Cell (DC) bridges the gap between experimental data and the *in-silico* simulations.
- Constructing the first large-scale logical model of Dendritic Cell
- Applications of the DC model in human immunology

## Introduction

Dendritic cells (DCs) comprise a diverse set of antigen-presenting cells that are responsible for the recognition of foreign and self-antigens and the subsequent regulation and initiation of specialized adaptive and innate immune responses (1, 2). *Via* pattern recognition receptors, DCs can sense a wide range of epitopes expressed by pathogens and damaged cells (3). The sophisticated ontogeny of DCs enables them to maintain tolerance in the presence of foreign and self-antigens or to initiate an inflammatory response (4–6). Striking the right balance to antigen response puts DCs in a critical pathway for disease management (7, 8). An insufficient immune response to an antigen can suppress downstream cell differentiation leading to an increased risk of infection and malignancy (9, 10). An over-reactive or chronic immune response, however, can lead to auto-immune diseases, allergies, and chronic inflammation (11, 12).

DCs mediate adaptive responses through cell-cell interactions (e.g., antigen presentation *via* the major histocompatibility complex (MHC) classes), the increase of co-stimulatory immune checkpoint ligands/receptors, and through the secretion of pro-and anti-inflammatory interleukins, growth factors, and chemokines (13, 14). Antigen recognition triggers a cascade of signaling pathways that switch the DC cellular state from tolerant (immature) to inflammatory (mature) (15, 16). DCs comprise three major subtypes with distinct immunogenicity and plasticity: conventional DCs (cDC1 and cDC2), plasmacytoid DCs (pDCs), and monocyte-derived DCs (MoDCs). As mature DCs, they can prime effectors and suppressors cells (e.g., lymphocyte T and B cells) to stimulate a wide range of immune responses (17, 18).

The significance of DCs in identifying and initiating an adaptive response to foreign and self-antigens has stimulated interest in isolating DCs as a potential therapeutic tool to program specific immune responses to pathogens and malignant cells (19–21). For example, in 2010, a DC-based vaccine was approved for the prevention of prostate cancer (22). DC-based vaccine development for other diseases has not been as successful; achieving full maturation of DCs and a limited ability for DCs to activate T cells are some of the challenges that have been encountered (23). Improved methods for characterizing and perturbing the complex mechanisms underlying DC maturation in the context of the broader immune system may help translate biological knowledge to clinical applications. Computational models, for example, have been gaining traction as a means to study the dynamics of immune responses in the context of homeostasis and diseased states (24–26) by utilizing a variety of mathematical frameworks (27–29) to represent multiple levels of biological regulation (e.g., genome-scale metabolic network regulation, signal transduction, cell-to-cell communication, etc.). Despite previous modeling efforts of immune-related biological diseases, a large-scale model of major DC functions and its communication with other immune cells is still lacking.

Multiscale models have the potential to uncover the underlying mechanisms behind emergent behaviors at various scales such as intracellular, cellular, and systemic levels. Such models can consider various temporal and organizational scales including signal transduction, gene regulation, metabolism, cellular behaviors, and cytokine transport (25). Different multiscale models have been developed to study the dynamic response of DC under different extracellular environments. For example, Klinke II DJ. developed a multiscale model to investigate the impact of the lung microenvironment on the education of DC for optimal T cell polarization. The model considered DC trafficking and education in the lung while taking into consideration the time, maturation, spatial distribution and IL12 response (30). Mei Y. and colleagues created a multiscale platform, the ENteric Immune Simulator (ENISI), to study the mucosal immune response during colonic inflammation. The multiscale tool has the advantage of connecting three different scales - intracellular, cellular, and tissular - using different mathematical languages (31). Lai et al. developed a multiscale model of DC-based vaccine by considering the signaling pathways underlying DC maturation, the bio-distribution of DCs in multiple organs, and the DC-T-cell response to identify optimal targets for enhancing anti-cancer DC vaccination in the context of melanoma (28). Although those multiscale models included a tissue scale to study the dynamic distribution of DC in different organs, so far, these models have only focused on DC functionality under specific disease conditions or specific signaling pathways. However, DC functions involve complex intracellular and cellular networks that are critical for regulating cell activation and initiating immune responses.

Logical modeling formalism has emerged as a particularly effective approach to modeling large-scale biological systems due to scalability and independence of kinetic parameters that are largely unknown (32–35). Logical models of different scales and complexity (a few to hundreds of components) have been applied to study various biological and translational questions (36), such as studying cellular crosstalk (37) and predicting cellular phenotypes (24, 38) and drug targets (39).

Here, we present a multiscale mechanistic model of human DCs that captures the complex interplay of intracellular molecular signaling to intercellular cell-cell communications. DC model enables researchers to easily modify, expand and test new hypotheses of the immune system. Our aim is to provide the researchers with computational tools to gain insight into DC mechanisms and disease pathology. The mechanistic model uses the logical mathematical framework (40) and focuses on signal transduction networks responsible for regulating DCs’ antigen-presenting cellular function, cellular interactions, maturation process, and immune cell population dynamics. It captures the dynamic biological events in response to diverse stimuli (pathogens, malignancy) and the downstream biological coordination between surface molecules (receptors, integrins, lectins), signal transduction (kinases, enzymes, transcription factors), and secretory factors (cytokines, chemokines). Two diseases are highlighted to demonstrate the utility of the model under diverse conditions. Lastly, receptor-ligand interactions between DCs and four immune cell types that DCs commonly interact with (T cells, B cells, natural killer (NK) cells, and neutrophils) have also been represented. The results of *in-silico* simulations of the model under various environmental conditions and network perturbations were validated using peer-reviewed published literature.

## Methods

### Model construction

The computational model is a mechanistic, logic-based model. Each component of the model can assume an active (1) or inactive (0) state at any time *t.* The activity state of the model’s internal components is determined by the regulatory mechanisms of other directly interacting components. These regulatory mechanisms are described with Boolean functions comprised of AND, OR, NOT operators (40).

To gain a comprehensive understanding of the molecular pathways involved in dendritic cells and antigen-presenting cells, we conducted a systematic search of the literature using PubMed. Our search was specifically focused on exploring the molecular pathways involved in each DC subtype: pDC, cDC1, cDC2, and MoDC. To limit the search results, we utilized a combination of search terms, including: “dendritic cells AND antigen-presenting cell AND MoDC AND molecular pathway,” “dendritic cells AND antigen-presenting cell AND pDC AND molecular pathway,” “dendritic cells AND antigen-presenting cell AND cDC1 AND molecular pathway,” and “dendritic cells AND antigen-presenting cell AND cDC2 AND molecular pathway.” This comprehensive search allowed us to obtain a wealth of information related to the molecular pathways involved in each DC subtype, providing a foundation for our investigation into the function and activation of these important immune cells. In the development of our model, we followed strict data inclusion criteria, limiting our selection to original research articles focused on healthy human subjects. Studies using mice and clinical trials were excluded from our manual literature mining process. The first draft of the model was constructed using the information obtained from the manual literature mining of the original studies. Upon reviewing the initial draft, we conducted a supplementary search of the literature utilizing both review and original studies to obtain well-established biological information related to regulators of the unconnected components. This thorough and systematic approach allowed us to develop a comprehensive model using 92 publications (83 original and 9 reviews) that represents the molecular pathways involved in dendritic cells. We defined subtype-specific markers to differentiate between pDC, cDC1, cDC2, MoDC (Results and **Supplemental Figure S1A**).

To validate the model, we collected literature reporting specific DC response to different extracellular conditions and simulated emergent behaviors that were not directly programmed into the model (41). Because logical models are of qualitative nature, model validations focus on the ability of the model to reproduce qualitative behaviors seen in wet-lab experiments (e.g., change in activity level of a component(s) under specific extracellular conditions) - a standard process for logical models (24, 41–43).

**Figure 1:**
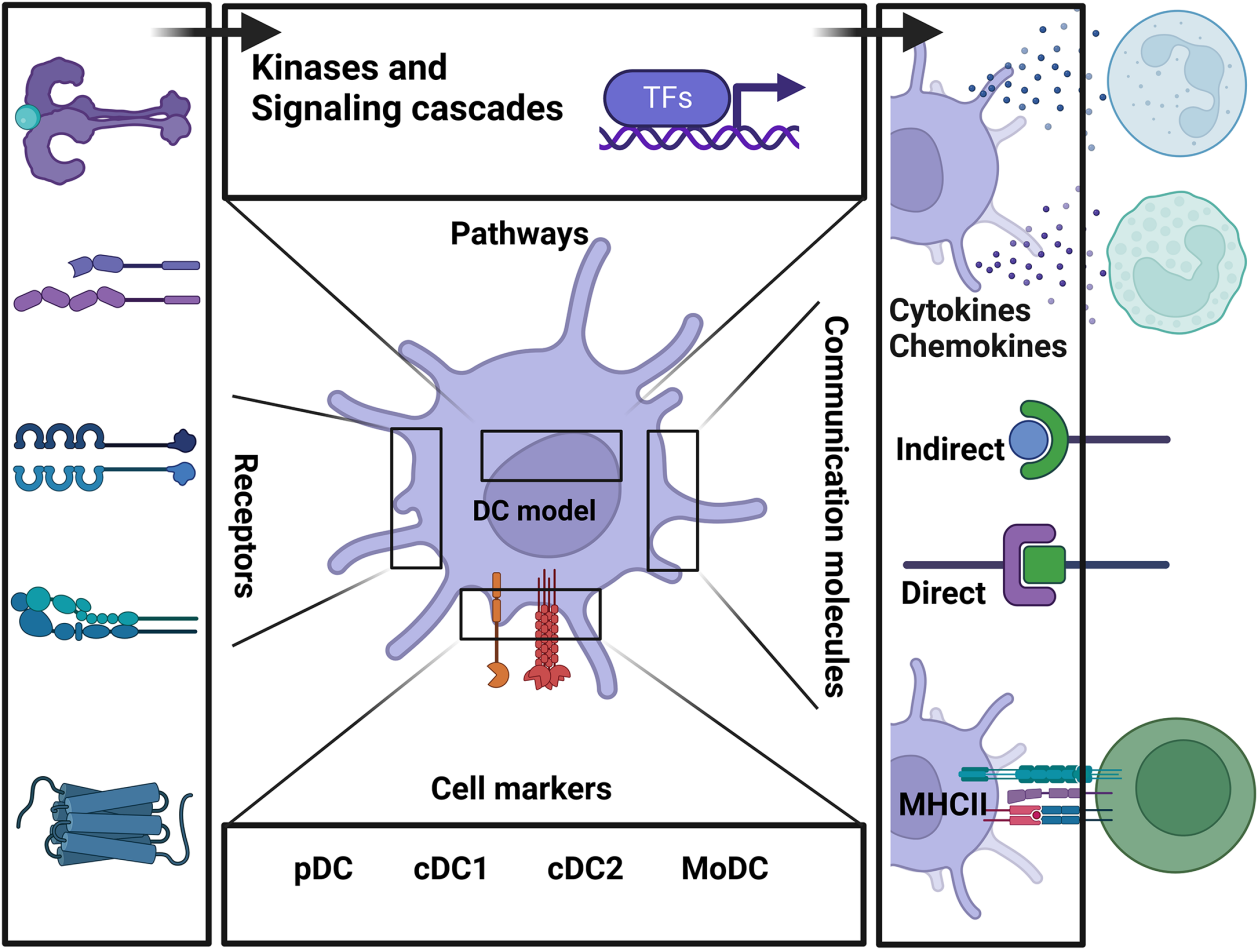
Schematic representation summarizing the main components of the model and the connection between each biological layer of the system.

From the publications, we retrieved information related to DC-specific stimuli, the effect of the studied environment, and comprehensive signaling pathways (receptors, kinases, transcription factors). The model consists of 281 components. These components are categorized into various classifications and compartments. There are 178 proteins, 87 RNAs, and 16 components representing phenotypes and cells. The proteins are organized in cell membrane (64 components), cytoplasm (40 components), nucleus (22 components), extracellular space (52 components). **Figure 1** Created with BioRender.com.

To standardize the naming convention of the components in the model, we used protein and gene names from the HUGO Gene Nomenclature Committee (HGNC) (44). The model was built in the web-based modeling and analysis platform, Cell Collective, and manually curated using the aforementioned literature (45). All components used to build the regulatory mechanisms have been annotated in Cell Collective with the exact quote from the reference literature. The model is publicly available in Cell Collective (under Published Models) where it can be simulated as well as downloaded (and other logical models published by the community) in several file formats (such as SBML-qual, text file of logical functions, and truth tables) (46, 47).

### Model simulations and analyses

Cell Collective was used to perform all computational simulations and analyses of the model. Cell Collective uses discrete mathematics to construct the model, but the simulated output values are semi-continuous, ranging from 0 to 100% activity levels (48, 49). The activity levels of external components are unitless and defined as a percent chance (probability * 100) of the component being active in a specific time *t* (24). Depending on the desired experiment, the activity levels of external components can be set by the user to specific values, or they can be set to ranges from which values during each simulation are selected randomly (e.g., to simulate dose-response experiments).

We used Cell Collective for two types of analyses: real-time and dose-response using asynchronous updates such that all genes take different times to make a transition, which is closer to biological phenomena (50). The initial condition of the model was set to immature cellular phenotype as 1 (active) and all other components were set to zero since DCs are considered immature under the resting condition and before stimuli activation (51). The immature DCs are recruited to the inflamed site by pathogen signals, capture foreign antigens and undergo maturation to DC subtypes (52). While simulating the model in Cell Collective, the user can define the activity levels of external components to a specific point or provide ranges (e.g., varying between 0% to 100%). When a range is defined for external components, their activity levels are selected randomly in each simulation. In the real-time simulation, we showed the activity of components at different times (steps), which was presented using the mean activity level of multiple simulations. For dose-response analysis, we conducted each simulation consisting of 800 steps. The activity levels output components are fractions of ones over the last 300 iterations (500 to 800 steps) that describe the model’s steady behavior as described by (48, 49). Under each environment set for a biological scenario, we used 1,000 simulations.

### Global sensitivity analysis

We used sensitivity analysis in Cell Collective to determine the association between external components (e.g., *in-vitro* inducers) and internal components (such as TLRs, cells, cytokines, and phenotypes). We used probabilistic global sensitivity analysis based on standardized regression coefficient (SRC) using the “sensitivity” package in R (24, 53) on input and output data of Cell Collective. In a single-input setting, we used SRC, which measures the strength of association between dependent and independent variables (53). We performed Cell Collective simulations under input activity levels ranging from 0 to 100%. The activity levels of inputs and outputs were independent and dependent variables in the statistical model. A higher SRC value represents a higher strength of association between input and output variables. We used SRC and k-means clustering algorithms (900 samples, specified three clusters for low, medium, and high activity levels) methods to visualize the simulation results.

### Kyoto Encyclopedia of Genes and Genomes pathways analysis

The Kyoto Encyclopedia of Genes and Genomes (KEGG) pathway (54) enrichment analysis was conducted using the DAVID Bioinformatics Resources (2021 Update) (55) to explore the model components at the functional level. DAVID is a gene functional classification tool in which we used a p-value <0.05 with a false discovery rate (< 5%) as the cutoff criterion for KEGG pathway enrichment. We used the ggplot2 R package to visualize the fold enrichment and P-values of the top 20 enriched KEGG pathways.

## Results

### A large-scale multicellular, mechanistic model of signal transduction regulation of dendritic cell immune responses

We constructed a mechanistic multiscale model of signal transduction networks governing the proper function of human DCs, spanning biological scales from molecular to cell-to-cell communication. The model comprises 281 components and 702 interactions between these components that regulate the DC responses. The multiscale nature of our model is based on the intercellular and intracellular communication dynamics between DCs and four other immune cell types, serving as a bridge between innate and adaptive immunity (56).

To facilitate the model’s utility, its architecture across various biological scales is first depicted in a biological illustration of the pathways, communication molecules, cell markers, and receptors involved in regulating DCs from immature cells to mature phenotypes (**Figure 1**).

**Figure 2:**
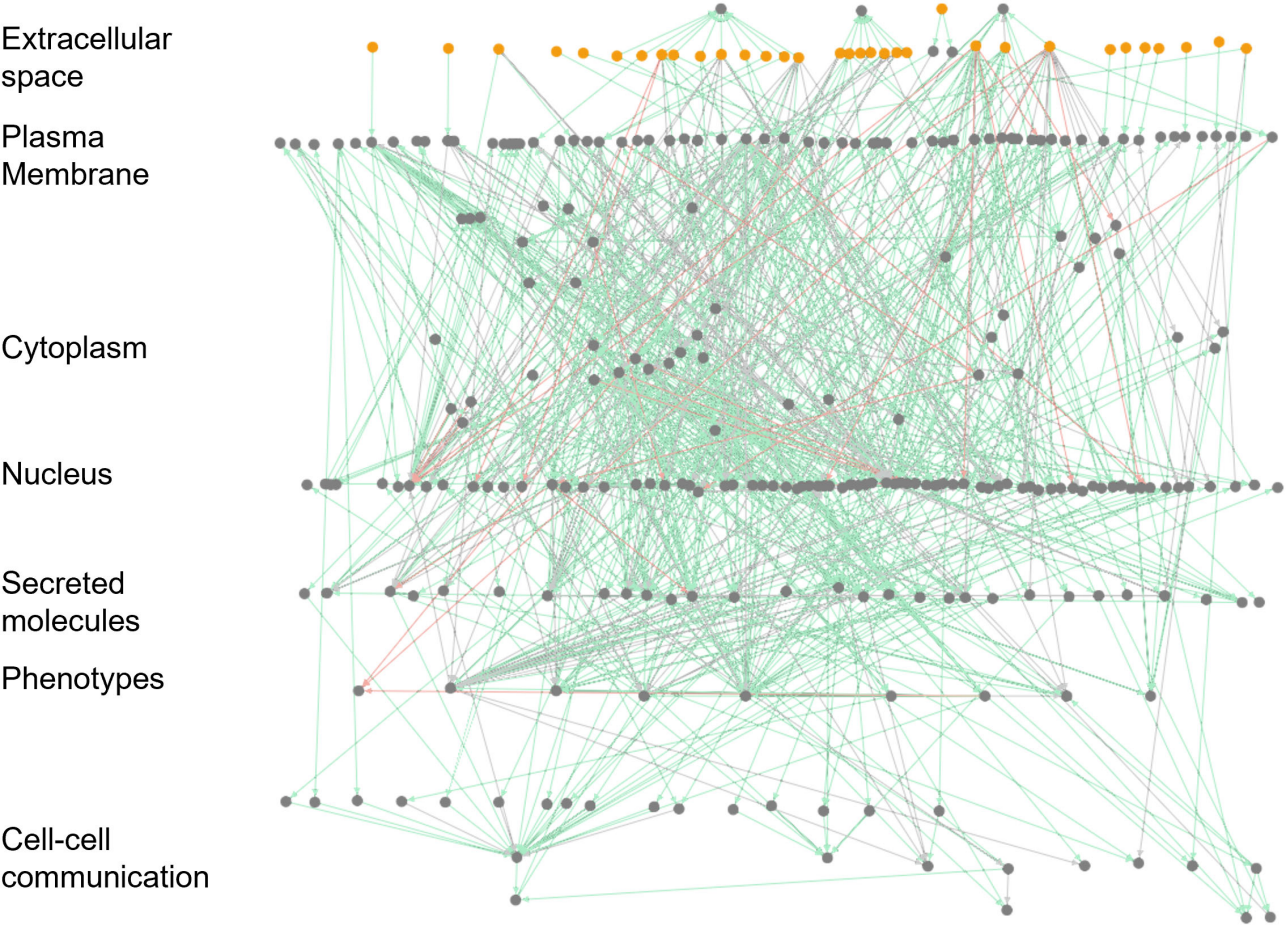
Visualization of the large-scale DC model in Cell Collective. The network view of the model. Dots represent signaling molecules; edges represent interactions between the model components. Red edges represent the inhibitions, while green and gray edges show activatory interactions. External (stimuli) and internal components are colored yellow and gray, respectively.

Further, the model’s architecture is depicted in **Figure 2**. Herein, the organization follows communication from the DC’s extracellular space to the plasma membrane (ligand-receptors, markers), to the cytoplasm (kinases and signaling cascades), to the nucleus (transcription factors and gene regulations) and to the secretory compartments that communicate with various DC phenotypes and other immune cell types that interact with DCs (**Figure 2**; **Table S1** ). The model is freely available in the Cell Collective modeling software and repository (45, 57) (DC model Link and workflow to Cell Collective environment in **Supplemental Figure S2**). Each component and interaction of the model have been fully annotated to facilitate data transparency and reusability. In Cell Collective, the community can also directly simulate and analyze the model, further improve it, or download it as an SBML file (46).

The model includes main signaling pathways, immune checkpoints, cytokines, and DC response mechanisms, and is able to represent the DC antigen presentation and maturation functions. The intra-cellular scale includes receptors that can sense the cellular environment and the downstream pathways that regulate DC responses that result in different cellular phenotypes. A diverse group of pattern recognition receptors (PRRs) is included in the model, including toll-like receptors (TLRs; TLR1, 2, 3, 4, 7, 8, and 9), C-type lectin receptors (CLRs; CLEC4C, CLEC7A, CLEC9A, CLEC10A, CD209), nucleotide-binding domain/leucine-rich repeat-containing receptors (NLRs; NOD2). The model also contains regulatory proteins (NF-κB, PYCARD, NLRP3, SYK, LILRA4, ISG20, ADAR, BST2, DDIT3, ATF4, PPP1R15A), maturation molecules (CD80, CD83, CD86, CD40, HLA-DR), and cytokines (IL6, TNF, IL12, IFNA1, IL12A, IL1B, IL12B, IL23A, IL10, IFNB1) (58).

In our study, we differentiated between various DC subtypes using a combination of subtype-specific markers. For pDCs, we employed CLEC4C (C-type lectin domain family 4 member C), and NRP1 (neuropilin 1) (59–61). For cDC1, we utilized CLEC9A (Dendritic cell C-type lectin receptor 9A), XCR1 (XC chemokine receptor 1), and THBD (thrombomodulin) (59, 60, 62–64). cDC2 was characterized using CLEC10A (C-type lectin domain containing 10A), and CD163 (CD163 molecule) (59, 60, 65). For MoDCs, we applied MRC1 (mannose receptor C-type 1), and CD1A (CD1a molecule) (59, 60, 66).

The intracellular molecular cascades stimulate interaction with other immune cells, including effector and exhausted T cells, B cells, NK cells, and neutrophils. For example, DCs capture and display antigen protein fragments on their plasma membrane through the antigen presentation process (67) then antigenic peptides are bound to appropriate molecules of the MHC, also known in humans as the human leukocyte antigen (HLA). T cells can recognize the antigens at the T cell-APC interface. DCs’ highly stimulatory and versatile APC function produces cytokines, interferons (IFNB), and tumor necrosis factor superfamily (TNF) to stimulate naive T cells to differentiate into effector subsets (68). As such, cytokines (IL1, IL6, IL10, IL12, IL23) and IFNB are also included in the model. DCs increase the expression of the MHC, the adhesion molecules, and the co-stimulators upon maturation, further stimulating T-cell proliferation and cytokine release (69). DC immune checkpoints are also included in the model (ICOS-LG, TNFSF9, TNFSF4, CD70, PVR, Nectin-2, BTLA, and PD-L1); the checkpoints regulate stimulatory and inhibitory pathways capable of maintaining self-tolerance and facilitating the immune response (70).

### *In-silico* model validation

To validate the mechanistic model of DC functions, we collected experimental data from 30 different studies (**Supplemental Data**, **Table S2**, **Figure S1**) and reproduced them *via in-silico* experiments. Below we describe four *in-silico* experiments that showcase how the model was used to validate well-known (extensively published) *in-vitro* experiments spanning intracellular communication dynamics, intercellular communication dynamics, and both inter and intracellular communication dynamics.

The first experiment assessed the model’s ability to reproduce the behavior of TLRs. Namely, Grandclaudon (26) studied a range of DC molecular states expressing various patterns of communication signals. The authors present that DCs were treated for 24 hours with lipopolysaccharide (LPS), which activated TLR4 signaling pathways and induced DC communication molecules, including IL1β, IL6, TNF-α, and IL12 cytokines. Similar studies (58, 71, 72) present the LPS-induced secretion of DC cytokines as a non-trivial test to investigate the TLR4 cooperation in response to infections. A better understanding of the mechanisms of host resistance can provide a basis for the development of more effective adjuvants and immunotherapeutic regimens.

**Figure 3:**
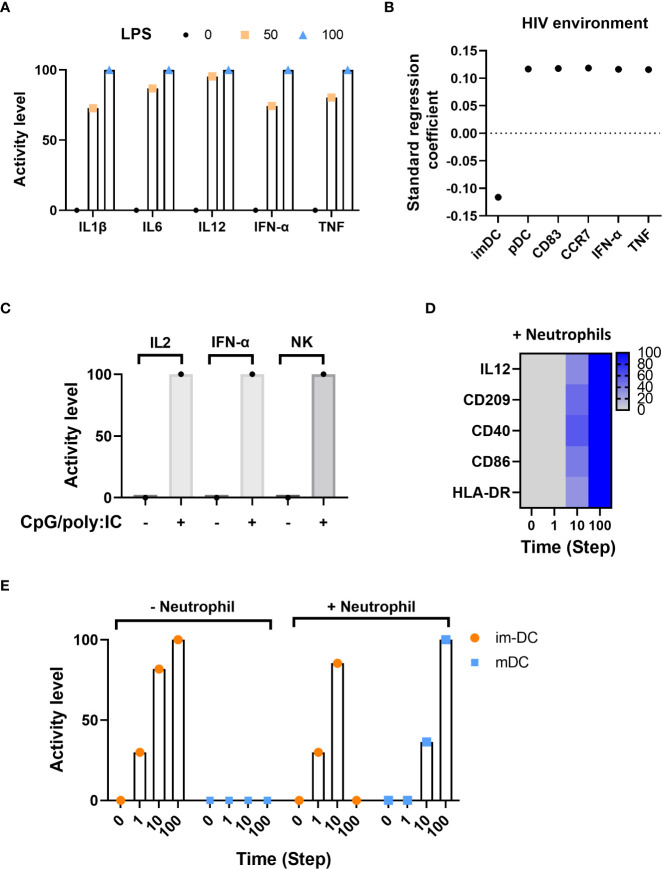
*In-silico* model validations. **(A)** Inflammatory cytokine activity level in response to LPS using dose-response analysis. **(B)** Standard regression coefficient of main factors activated in the HIV infection environment. **(C)** Activity level of IL2, IFN-α and NK under CpG-containing oligonucleotides and poly(I:C) stimulation. **(D)** Markers of maturation at different time points in the presence of neutrophils. **(E)** Time course distribution of DC immature (im-DC) and mature (mDC) states with (+) or without (-) neutrophils.

To validate the ability of the model to reproduce TLR behavior, we first validated that the model contained all components and regulatory pathways to support this experiment. Next, we ran 1,000 dose-response simulations while defining three activity levels of LPS (0, 50, and 100). We then evaluated and compared the secretion of inflammatory cytokines – IL1β, IL6, IL12, IFN-α, and TNF – upon activation by LPS at each of the three different dose responses against well-established cytokine responses. **Figure 3A** displays the secretion level for each of the five cytokines at LPS doses 0, 50, and 100. As expected, there was no cytokine secretion at dose 0 and subsequent secretion and elevated secretion at doses 50 and 100, respectively.

The second experiment assessed the model’s ability to mount an appropriate immune response to the presence (and initiation) of the human immunodeficiency virus (HIV) infection. HIV initiates viral transcription through TLR8 and promotes the maturation of DCs (from immature (imDC) to mature plasmacytoid (pDC)) as defined by the expression of CD83 and CCR7 surface markers and the production of IFN-α and TNF (73, 74). We used Cell Collective’s global sensitivity analysis (refer to the “Methods” section, “Global sensitivity analysis”) as a method to determine the association between HIV and each of the internal components. **Figure 3B** displays the correlation of activity for imDC, pDC, CD83, CCR7, IFN-α, and TNF in the presence of HIV infection. As expected, immature DCs (imDC) exhibit a negative correlation in the presence of HIV, which shows HIV-bearing immature DCs can differentiate into mature DCs in response to the infection, presenting HIV antigens to T cells and initiating viral immune responses. Further, mature DCs (pDC), as well as surface markers CD83 and CCR7, and IFN-α and TNF exhibit a positive standard regression coefficient (SRC), which means an increase in HIV load results in increased activity of these components. The simulation results are consistent with biological experiments that describe pDCs exposed to HIV strongly upregulating the expression of CD83 and functional CCR7 maturation markers, IFN-α, and TNF cytokines (73).

The third experiment assessed the model’s ability to simulate known intercellular dynamic crosstalk between DCs and other immune cells in tandem with intracellular communication dynamics. Gerosa (75) showed that human peripheral pDC and MoDCs are necessary to induce NK cell function depending on the type of microbial stimulus. In this experiment, pDCs and MoDCs were stimulated in response to CpG-containing oligonucleotides (CpG) and poly(I:C), and evaluated the mean activity level for NK cells, IL2, IFN-α, as a result of (CpG)/poly(I:C)-induced release of IL2 and IFN-α and subsequent activation of NK cells. **Figure 3C** displays the expected activity of IL2 and IFN-α as well as NK cells when CpG/poly(I:C) is inactive compared to an active state.

Last, we validated intercellular communication dynamics between imDCs and neutrophils. Neutrophils stimulate imDCs to become competent antigen-presenting cells. This maturation phenotype is characterized by the expression of specific surface markers (e.g., HLA-DR, CD86, and CD40) and the secretion of IL12 in response to DC-neutrophil interactions (76–78). **Figure 3D** displays the activity of model components IL12, CD209, CD40, CD86, and HLA-DR in response to the presence of neutrophils over time, demonstrating the pathways responsible for neutrophil-induced DC maturation. **Figure 3E** shows the activity level of immature and mature DCs in the presence and absence of neutrophils. On the left, when neutrophils are absent, immature DCs continue to increase in activity over time, whereas mature DCs do not become active. On the right, as neutrophils become present, immature DC activity tapers, and mature DC activity increases.

The aforementioned experiments illustrate the ability of the model to reproduce major experiments spanning complex inter- and intracellular communication dynamics.

## Case studies

To aid researchers in identifying how to use this model, we showcase three case studies by presenting a brief application background, the method we used to apply the model in this context, and model results.

### Case 1: Intracellular communication dynamics. Characterization of DC response to a combinatorial COVID-19 and Influenza infection environment.

In this case study, we integrated Covid-19 and Influenza pathogens into the model to characterize the molecular response of DC under single and co-infection conditions. Coronavirus disease 2019 (COVID-19) and Influenza respiratory disease, caused by Sars-CoV-2 and influenza virus, respectively, share similarities in seasonal manifestations, viral transmission method, symptoms, and immunopathogenesis (79, 80). Co-infection with Sars-CoV-2 and influenza virus increases disease severity and impairs neutralizing antibody and CD4+ T cell responses (81). Patients can develop both infections, and in some cases, co-infection leads to a poor prognosis (82–84). Despite the comprehensive investigation of DC behavior in single infections with Sars-CoV-2 or influenza, the comparative understanding of DC programming under co-infection is not fully explored due to limited patient cohorts and case studies (85–88).

**Figure 4:**
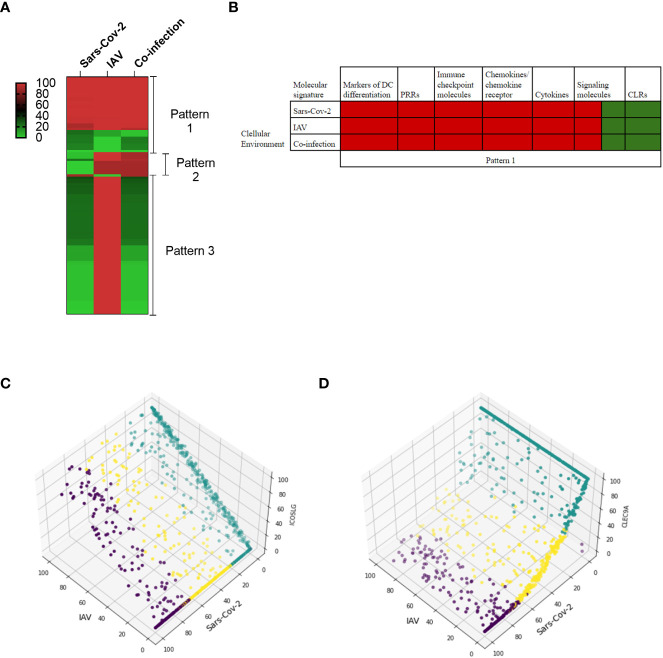
*In-silico* predictions of molecular activity across the whole DC model comparing Sars-CoV-2 and Influenza A virus (IAV) co-infection to the single infection. **(A)** The differential molecule expressions are grouped into three main patterns in response to each environmental setting. The first pattern grouped molecules that are regulated similarly in all three conditions. The second pattern is related to similar regulation between Influenza and co-infection, and the third one grouped similar behavior between Sars-CoV-2 and co-infection conditions. The scale represented the activity level ranging between 0 to 100%, 100 being the highest activity level. **(B)** The molecular signatures in pattern 1 in single and co-infection cellular environments. **(C)** An example of the second pattern shows that under different activity levels of Sars-CoV-2 (green, low; yellow, medium; purple, high), ICOSLG is inactive while it is upregulated in both co-infection and single IAV. **(D)** CLEC9A is categorized as the third pattern, and for both Sars-CoV-2 and co-infection, it has low activity levels compared to the IAV single infection.

Thus, the purpose of this model-based study was to investigate the molecular behavior of DCs in three infectious states: infection with i) Influenza type A virus (IAV), ii) Sars-CoV-2, and ii) co-infection with IAV and Sars-CoV-2. We ran 900 dose-response simulations and identified three molecular patterns (**Figure 4A**, **Table S3**) based on similarity in activity levels of DCs molecular components. Each pattern presents a list of molecules that play a role in the co-infection. We reported the mean activity level of DC model molecules ranging from low activity (green) to fully activated (red) molecular state in single infection and co-infection cellular environments.

In the first pattern (**Figure 4A** – Pattern 1 and **4B**), we identified molecular signatures with similar activities in single infection and co-infection. **Figure 4B** presents molecular signatures following this pattern, including markers of DC differentiation (CD86, CD1A, CD40, CD83, ITGAM), PRRs (TLR8), immune checkpoint molecules (PVR, Nectin2), chemokines/chemokine receptor (CCR7, CCL19, CXCL8), cytokines (IL6, TNF, IL12, IL1B, IL12A, IL12B, IL23, IL10, IFNA1, IFNB1), signaling molecules (NF-κB, PYCARD, NLRP3, SYK, LILRA4, ISG20, ADAR, BST2, DDIT3, ATF4, PPP1R15A), and CLRs (CLEC4C, CLEC10A).

Several of these signatures are expressed during the single infection studies on human samples (89–91). For example, separate studies on Sars-CoV-2 and influenza virus infections show expression of IL1B, IL10, TNF, CD86, CCR7, IL6, CXCL8, IFN (79, 89, 92, 93).

In the second pattern (**Figure 4A** – Pattern 2), the molecular signature characterizes the similarity between IAV single infection and co-infection. Previous studies indicated that immune checkpoints are increased in influenza single infection (94) but not in Sars-CoV-2 single infection (89, 95, 96). Thus this experiment focuses on the significance of the immune checkpoint signatures. The immune checkpoints (TNFSF4, CD70, ICOSLG, PDCD1LG2), followed by cytokines (IL2, IFNL2, CXCL10), markers of DC differentiation (CD80, CD86), and signaling signature (SEMAD4), are upregulated in both co-infection and single IAV but downregulated under Sars-CoV-2 infection. As an example, **Figure 4C** shows the activity level of the ICOSLG immune checkpoint in 300 simulations per each infection condition (single and co-infection), which is higher in the presence of both viruses.

In the third pattern (**Figure 4A** – Pattern 3), the molecular signatures of Sars-CoV-2 and co-infection were similar. The major signature includes a decreased expression of signaling and decreased expression of transcription factors in both Sars-CoV-2 and co-infection, suggesting a disruption of the signaling network associated with Sars-CoV-2 infection. Neuropilin-1 (NRP1), the only signaling protein to be highly expressed in the third pattern, was previously shown to facilitate Sars-CoV-2 entry by interacting with spike protein (97, 98). Additional signatures are related to the decrease of pathogen sensors and maturation marker expressions, such as TLRs (TLR1, TLR7, MYD88), CLRs (CLEC9A, CLEC7A, CD209), and MHC class signatures (HLA-DQA, HLADPB1, HLA-DM, HLA-DRB1, HLA-DR, HLA-DQB1), suggesting the loss of DC function to sense and present antigen to other immune cells properly. For example, **Figure 4D** presents the simulation results of the C-type lectin domain containing 9A (CLEC9A) with a low activity level in co-infection.

Several studies indicated that DCs displayed a defect in maturation and are depleted in COVID-19 patients, and as our *in-silico* simulations predicted, one of the mechanisms might be due to the defect of the signaling compartment and pathogen sensors (85, 86, 99). Nevertheless, further experimental investigations are needed to explore these hypotheses.

### Case 2: Intercellular communication dynamics. Crosstalk between DCs and T cells in a cancer microenvironment.

DCs play a crucial role in initiating a protective anti-tumoral response by presenting tumor antigens and providing co-stimulatory immune checkpoint to T cells (100). However, tumor microenvironments sustain DCs in an immature/tolerant phenotype, thereby altering antigen presentation, co-stimulatory signals, and thus the ability to effectively activate T cells. Therefore, T-cells become exhausted due to continuous exposure to antigens and increase in multiple inhibitory immune checkpoints that further benefit the mechanism of resistance to immunotherapies (101).

Several factors with immunoregulatory properties are involved in DC-T cell interplay. For example, the cytokine HMGB1 released by cancer cells contributes to cancer development by promoting tolerogenic DC differentiation and the suppression of anti-tumoral T cells (102–104). Moreover, a study conducted *in-vivo* reported the role of HMGB1 in promoting T-cell exhaustion in the condition of trauma (105). However, the role of cancer-derived HMGB1 in promoting exhaustion through the modulation of immune checkpoint expression has not been investigated. Modern immunotherapy approaches aim to reverse T cell exhaustion by blocking inhibitory immune checkpoint receptors (106). Combinatorial treatments using approved inhibitors of PD-L1 immune checkpoint and two receptors PD-1 and CTLA-4, showed promising results. Additional immune checkpoint inhibitors are under clinical trial investigations (107). However, not all cancer types respond equally, and patients can acquire resistance to immune checkpoint inhibitors (ICI) (108, 109). Because many experimental studies have investigated the role of immune checkpoints individually, a computational approach can help to better understand the dynamic distribution of inhibitory and stimulatory immune checkpoints that can aid in identifying ideal checkpoint candidates and facilitate combinatorial therapeutic strategies.

**Figure 5:**
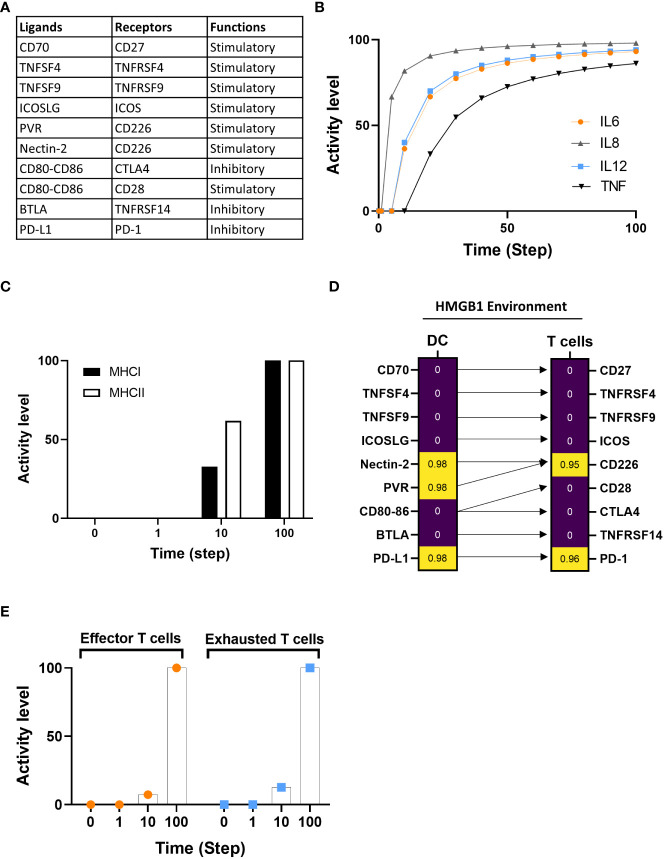
Investigation of DCs-T cells crosstalk under an HMGB1 tumor environment. **(A)** The table indicates the immune checkpoint pairing between ligands and associated receptors along with the type of functions (stimulatory or inhibitory). **(B)** Assessment of IL6, IL8, IL12, and TNF cytokine expression under HMGB1 environment. **(C)** The activity level of MHC classes I and II are between 0 and 10, with the time 100 added at the final expression of both classes. **(D)** Standardized regression coefficient (SRC) between ligands and associated receptors on DC and T cells with HMGB1 environment. High and low SRC presented with yellow and purple, respectively. Arrows link the ligands to their respective receptors. **(E)** Time course of effector T and exhausted T cells activity level expression at a probability of activation at time steps 0, 1, 10, and 100 in HMGB1 simulation.

In this case study, we examined the impact of cancer-derived HMGB1 on the DC-T cell synapse interaction. We included the cancerous cytokine HMGB1 as environmental (cancer) stimulus. The DC model includes two groups of immune checkpoints: stimulatory and inhibitory ligands/receptors that are enable us to study the impact of HMGB1 on DC-T immune checkpoints. The ligands are expressed on DCs, while receptors are particularly expressed by T cells. In the model’s plasma membrane compartment (**Figure 5A**, **Table S1**) are included six stimulatory ligands (e.g., ICOS-LG, TNFSF9, TNFSF4, CD70), two inhibitory molecules (PD-L1, BTLA) and three molecules with a dual function depending on the receptors they are binding (CD80-CD86, PVR, and Nectin-2). From the T cell side, we included six stimulatory receptors (CD28, ICOS, TNFRSF9, TNFRSF4, CD27, CD226) to define effector T cells and three inhibitory receptors (PD-1, TNFRSF14, CTLA-4) that define exhausted T cells. **Figure 5C** showed the interaction between ligands with their respective receptors. Of note, CD80-CD86, used as main maturation markers, binds two different immune checkpoint receptors with opposite functions (the stimulatory receptor CD28 and the inhibitory receptor CTLA4), and the two ligands PVR and Nectin-2 share the same stimulatory receptor CD226 (**Figure 5A**).

We compared the model’s simulation results under the HMGB1/cancer environment with published experimental data (110). Messmer D. et al. showed that HMGB1 promotes the secretion of inflammatory cytokines (IL6, IL12, IL8, TNFα) and our *in-silico* simulation is consistent with the experimental data (**Figure 5B**). Then, in the HMGB1 environment, we evaluated: i) the activation of MHC Class I and II, ii) the distribution of ligands/receptors for co-stimulatory and inhibitory immune checkpoints expressed at the DC-T cell interface, and iii) the dynamic distribution of effector and exhausted T cells.

We evaluated the distribution of the mean percentage activity level of MHC class I and II in five *in-silico* experiments at time steps 0, 1, 10, and 100. The mean activity level started from 0, and at time step 10 reaches 32.74% for class I and 61.8% for class II. MHC class I and II increase to maximum activity level at step 100 (**Figure 5C**). Our simulation indicated that MHC class I and II expression increased in response to HMGB1 simulation. Because our model does not include specific tumor antigens that can be presented by MHC to TCR, we cannot conclude that the increase of MHC expression is due to antigen overload, however, our simulation indicates an increase of MHC classes under HMGB1 simulation.

Next, we investigated the dynamics of immune checkpoint pairs under HMGB1 environmental stimulation using dose-response and sensitivity analyses (**Figure 5D**). Our *in-silico* results showed that the stimulatory molecules such as CD27 (receptor for CD70), ICOS (receptor for ICOS-LG), TNFRSF4 (receptor for TNFSF4), and TNFRSF9 (receptor of TNFSF9) showed no significant correlation with HMGB1 stimulation and shared similar distribution with their receptors expressed by T cells. The PVR and Nectin-2 displayed high correlation as well as their receptor CD226. CD80-CD86 showed no significant correlation in response to HMGB1 stimulation (**Figure 5D**). Regarding the dual receptors of CD80-CD86, the stimulatory CD28 receptor, and the inhibitory receptor CTLA-4 showed no correlation under HMGB1 stimuli. The inhibitory pairing PD-L1 ligand with its receptor PD-1, the main target for immune checkpoint inhibitors, is highly represented. Moreover, the additional inhibitory receptor TNFRSF14 and its ligand BTLA ligand don’t show a significant distribution in response to HMGB1 stimulation (**Figure 5D**).

We simulated the model under HMGB1 environment, and we evaluated the mean activity level of effector and exhausted T cells at time 0’, 1’, 10’, and 100’ from five *in-silico* experiments (**Figure 5E**). The exhausted T cell activity level is faster than the effector T cells at 10’ (**Figure 5E**). At the maximum time of the simulation, both T cell phenotypes do reach maximum accumulation (activity). The results demonstrate HMGB1 promotes both effectors and exhausted T cells and exhausted phenotype accumulated faster than effector.

Immune checkpoint immunomodulatory functions are initiated by ligand-receptor interaction that can either promote or suppress T cell function (111). CD226 is important in generating an anti-tumor response. While CD226 expression is required as a co-stimulatory factor for T cells during antigen presentation by APCs, the loss of CD226 can lead to impaired effector T activation and increased susceptibility to tumor development (112–114). Hence, in our model, the activity of CD226-PVR/Nectin2 contributed to the increase of effector T cells and is associated with the MHC expression. Among inhibitor pairs, only PD-L1-PD-1 displays a high correlation with an HMGB1 simulation. The interaction between PD-L1 and PD-1 drives T-cell dysfunction and exhaustion to prevent an efficient anti-tumor T-cell response (115, 116). Previous studies indicated that HMGB1 increases PD-L1 expression in cancer cells; however, the modulation of PD-L1-PD-1 by HMGB1 in immune cells remains unknown. Our *in-silico* simulation suggests that HMGB1 can also promote PD-L1-PD-1 expression at the DC-T cell interface, thereby explaining the increase of exhausted T cells.

In summary, using the example of analysis of multiple ligand/receptor-mediated cellular programming at the time, our *in-silico* experiments illustrated the capacity of the model to provide complex and dynamic insight into biological processes at the molecular and cellular scales.

### Case 3: The scope of the DC models offers potential applications in several immune-related diseases.

The crosstalk between the disease environment and DCs highly contributes to the organization of the immune response (11, 12, 117–119). Because each disease environment is unique and complex, a multiscale model can be an effective tool to investigate the complexities underlying multiscale, systemic diseases.

**Figure 6:**
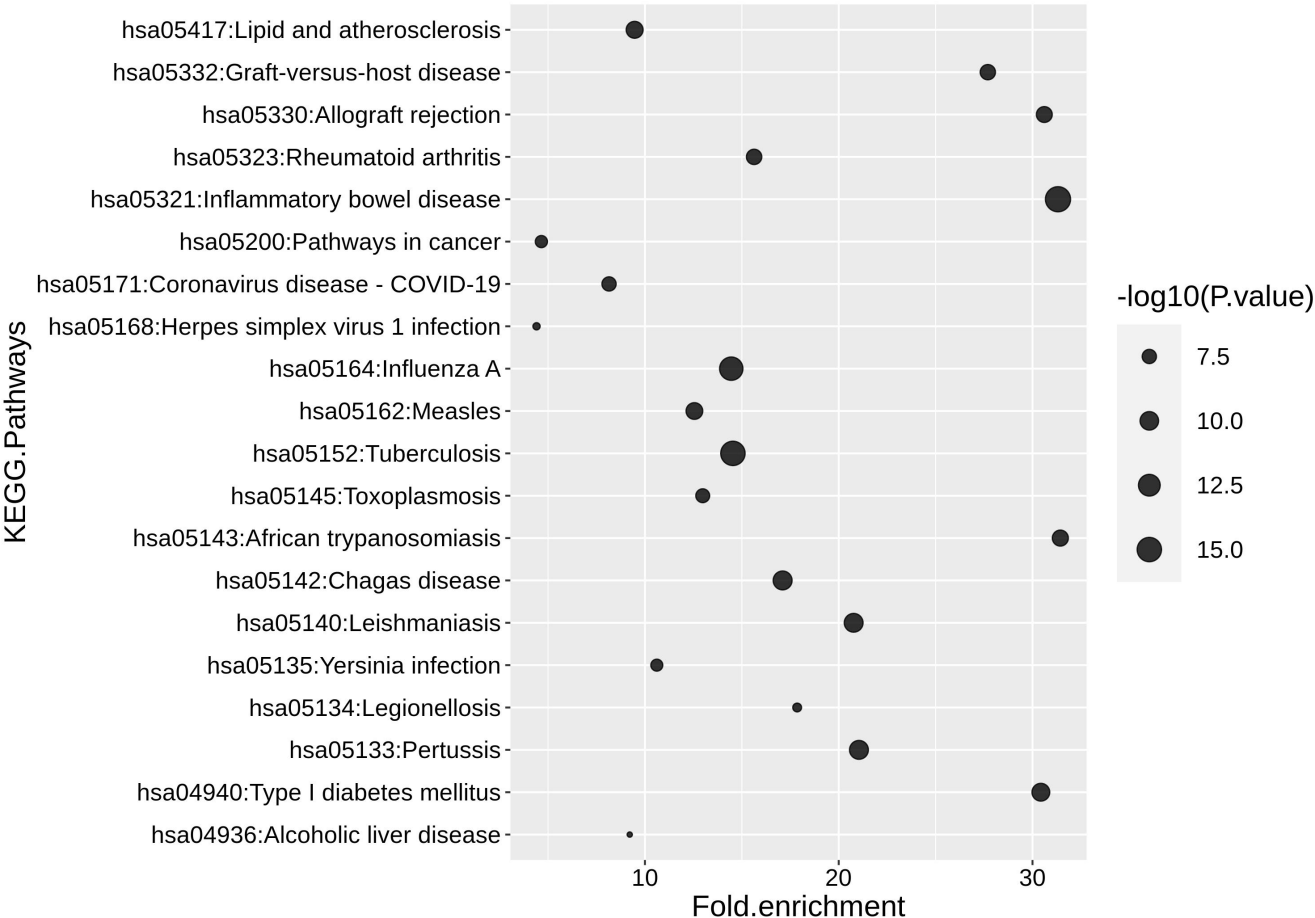
The top 20 human diseases and signaling pathways associated with the DC model. *P*-values and fold enrichment of KEGG pathways in DC. -log 10 of P-values was used for visualization. Thus, larger dot sizes correspond to lower *P*-values. The cutoffs *P*-value < 0.05 and a false discovery rate (FDR) < 5% were set for significant enrichment.

Given the DCs’ role in initiating both innate and adaptive immune responses, we sought to explore the links between the DC model’s core disease pathways we identified for IAV, Sars-CoV-2, and tumor microenvironment and additional diseases to identify the extensibility of our model. To do this, we performed a Kyoto Encyclopedia of Genes and Genomes (KEGG) enrichment analysis against the model’s components (refer to “Methods” section, “Kyoto Encyclopedia of Genes and Genomes KEGG pathways analysis”). Using a cut-off p-value of <0.05, we identified 69 enriched pathways (**Table S4**) for the top 20 diseases (**Figure 6**). The fold enrichment analysis of the top 20 diseases revealed multiple categories, such as autoimmune disease, infection, and transplantation.

The two highest scores are represented by inflammatory bowel disease and tuberculosis infection. The enrichment for those two diseases can be explained by the presence of TLRs, lectins, and cytokines in supporting pathology (120). For example, in the context of tuberculosis, TLRs and lectins can recognize different motifs of Mycobacterium tuberculosis, which in turn can trigger pro- or anti-inflammatory cytokine response (121).

The DC model can be utilized to identify pathways related to many diseases, including infections, cancer, and autoimmune diseases. The high fidelity of the model predictions will depend on the extension of the pathways related to the diseases of study.

## Discussion

We have developed a mechanistic multiscale model of human DCs that spans biological scales from molecular interactions to cell-cell communication. We included biological events that occur between DCs’ environmental stimuli and their receptors, followed by activation of signal transduction in response to each signal. Moreover, we constructed the molecular network that links the downstream signal transduction of kinases and transcription factors to secreted cytokines/chemokines and growth factors. We extended the model further by integrating a cellular compartment that includes the communication between DCs and several innate/adaptive immune cells through direct (ligand-receptor) and indirect (cytokine, chemokines releases) interactions. Our model can be used to study DC maturation, differentiation to each subset, APCs function, and the bidirectional crosstalk between DCs and other immune cells. Because the model incorporates pathways that regulate and facilitate many key functions of DCs, it can be applied to study several diseases as well as the basic mechanism of DCs’ functions.

The presented DC model leverages the widely used logical modeling formalism (40). The advantages of this modeling approach include its scalability (efficient simulations) as evidenced by the fact that some of the largest computational models have been constructed using this formalism (e.g (122–125). Another advantage is that logical models do not rely on kinetic parameters that are mostly unknown (40, 126). On the other hand, if one is interested in modeling relatively small and well-studied pathways (with known parameters), a kinetic modeling approach may be more appropriate. The model is limited by the missing data in the literature about any unknown interactions. Our model includes major pathways involved in DC immunobiology. Nevertheless, the model is limited in scope as it does not include all known DC signaling and cell-cell communication. The model is being provided in a readily exchangeable format (SBML) and easy-to-use modeling software (Cell Collective), making it relatively easy for the community to build on the model and continue to expand as needed by different applications. For example, to specifically investigate DC-T cell communication, T cell subsets such as CD4 and CD8 can be integrated by adding molecular and cellular components of the immunological synapse. We previously published a logical model of signal transduction networks governing CD4+ T cell differentiation in response to various cytokines (24). Those same cytokines are also included in our DC model, creating the possibility of integrating both systems to study how DCs might influence CD4+ T cell fate and plasticity.

As another example, HMGB1 interacts with several TLRs (e.g., TLR2, TLR4, and TLR9), which have been included in the DC model. HMGB1 also interacts with RAGE - a receptor for advanced glycation end-products - that is not currently included in our model. Adding RAGE to the system would increase the complex interplay between receptors and signaling pathways to mediate cytokine release and immune response (127–129). The model would then be able to simulate the different molecular intersections during single or multiple TLRs/RAGE activation and predict the multiple environmental conditions for efficient DCs maturation without compromising the adaptive response (e.g., T and B cells). Therefore, the multiscale model could be further used to characterize APC function in response to a stochastic tumor micro-environment with multiple components simultaneously.

In our cancer *in-silico* simulations, our model-generated hypotheses suggested a list of potential immune checkpoints to explore for studying the effect of single and multiple combinatorial ICI on DC-T cell interaction outcome (**Figure 5D**). We showed the dynamics of immune checkpoint pairs under a tumor HMGB1 environment. Recent therapeutic approaches include the optimization of DC-based therapies by combining DC vaccines with immune checkpoint inhibitors (ICI), such as anti-CTLA-4 and anti-PD1/PDL1 (130, 131), or by silencing immune checkpoint signaling pathways (132). Despite being in early clinical phases, combinatorial therapy holds a potential to balance toxicity, safety, and clinical outcomes (130, 131). Additional ICI to restore T cell or APC activation is currently under investigation to expand therapeutic options and optimize the efficacy of the immune checkpoint targeting strategy (107, 133). Nevertheless, the complexity of immune checkpoint ligands resides in their capacity to bind several different receptors with opposite functions, therefore switching between stimulatory and inhibitory signals. As the model prediction suggested, PVR and Nectin-2 showed a high activity similar to their receptor, CD226. Of note, PVR and Nectin-2 can trigger opposite signals whether they bind the stimulatory receptor (CD226) or the inhibitory receptors (TIGIT and CD96, not included in the model) (134). Moreover, the optimal combination can depend on the ligands/receptors’ availability and the balance between stimulatory and inhibitory expression. Our model simulations suggest that the inhibitory receptor CTLA-4 has no activity under HMGB1 stimuli. At the same time, PD-1 and PD-L1 are highly correlated, suggesting that the use of anti-CTLA-4 might not be as effective as the use of anti-PD-1 or anti-PD-L1 to restore DC-T cell function in a cancerous HMGB1 environment (135).

The development of computational models that recapitulate complex human disease behavior can be a resource for scientists and clinicians to simulate thousands of possibilities for studying the complex biological process at multiple scales. The disease enrichment analysis highlighted the potential of our model to incorporate additional pathological events as some disease modules are already implemented. For example, Type I diabetes (T1DM), an auto-immune disease characterized by immune-mediated destruction of insulin-producing beta cells, is enriched in our model (136). The loss of tolerance to self-antigens and the increase of autoreactive T cells instead of immunosuppressive T cells are the main cause of insulin deficiency. Several studies indicated that DCs presented self-antigen generated from degraded b-islet to prime autoreactive T cells *via* dysfunctional NF-κB and MAPK pathways (137, 138). Current therapies focus on generating tolerant DCs and immunosuppressive T cells to target the auto-immune disease and restore the imbalance of tolerance (139). To address those mechanisms, incorporating tolerogenic DCs and immunosuppressive T cell phenotype components under the stimulation of a self-antigen input could predict molecular conditions by which immunosuppressive cells are amplified to respond to disease pathology (140).

In summary, we have demonstrated the potential for a multiscale DC model to investigate the immunobiology of DCs and identify potential targets for improving the effectiveness of DC-based cell therapies. Lastly, the model can be further expanded to support additional mechanistic and therapeutic questioning related to DC ontogeny.

## Data availability statement

The original contributions presented in the study are included in the article/**Supplementary Material**. Further inquiries can be directed to the corresponding authors.

## Author contributions

SSA, RA and TH conceived the study. SSA, RA and TH designed the study. SSA performed literature mining and collected the data. SSA constructed the models. SSA, and RA performed refinement of the constructed models. SSA, RA and BLP analyzed the data, performed the experimental work and analyzed the experimental results. SSA, BLP, RA and TH wrote the manuscript. RA and TH supervised the study. All authors contributed to the article and approved the submitted version.
