# Supplementary material for: A multiscale mechanistic model of human dendritic cells for in-silico investigation of immune responses and novel therapeutics discovery
Source: Front Immunol. 2023 Mar 10;14:1112985. doi: 10.3389/fimmu.2023.1112985 (PMC10040975; doi:10.3389/fimmu.2023.1112985)
Supplement: Supplementary file 1 [file DataSheet_1.pdf]

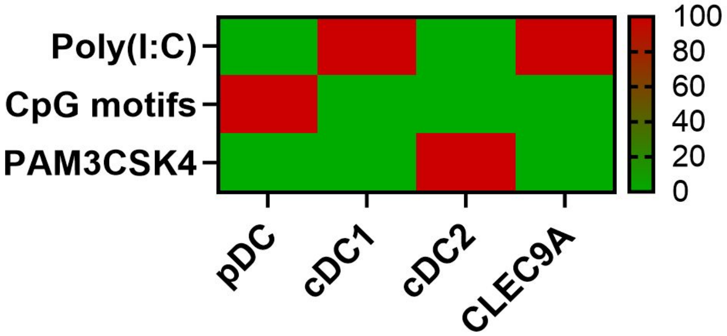

A)

Expression of CLEC9A in cDC1, pDC, and cDC2 was studied under different environmental conditions of Poly(I:C), CpG motifs, and PAM3CSK4 (*Macri et al., 2018*). The mean activity levels of the selected components after simulation were visualized with red and green to indicate high and low expression respectively. Our results showed that cDC1 expressed CLEC9A in the presence of Poly(I:C), while the CpG motifs and PAM3CSK4 conditions upregulated pDC and cDC2, respectively, but did not activate CLEC9A.

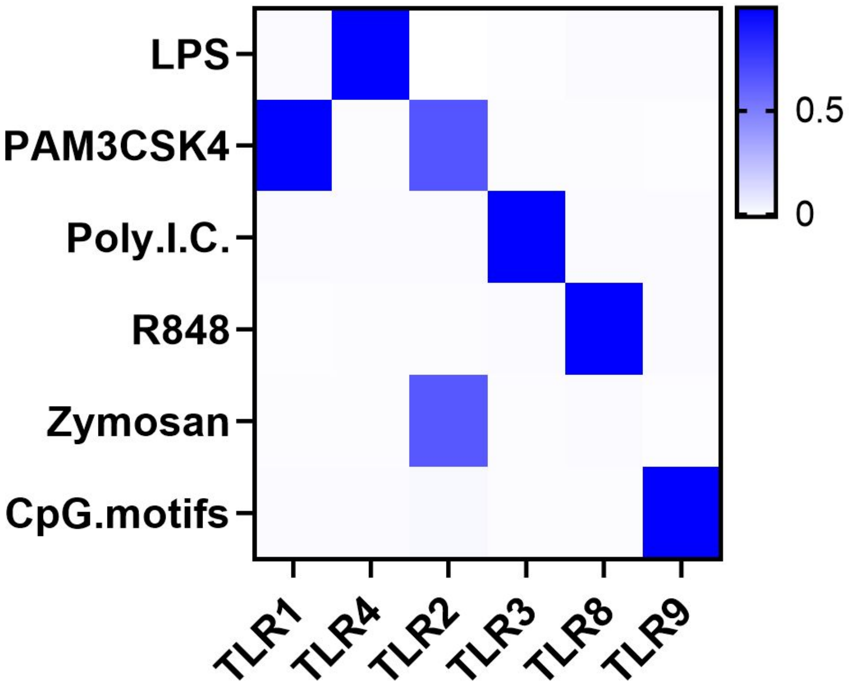

B)
Standard regression coefficient of TLRs activated in the in-vitro inducers environment. Our *in-silico* simulation associated adequate TLRs with their respective ligands, showing a significant positive correlation for TLR1-PAM3CSK4, TLR2-PAM3CSK4 and Zymosan, TLR3-Poly(IC), TLR4-LPS, TLR8-R848, and TLR9-CpG which is in agreement with experimental data (*Yu et al., 2010*).
