# Supplementary material for: A multiscale mechanistic model of human dendritic cells for in-silico investigation of immune responses and novel therapeutics discovery
Source: Front Immunol. 2023 Mar 10;14:1112985. doi: 10.3389/fimmu.2023.1112985 (PMC10040975; doi:10.3389/fimmu.2023.1112985)
Supplement: Supplementary file 2 [file DataSheet_2.pdf]

A) The link to the Demo of Cell Collective

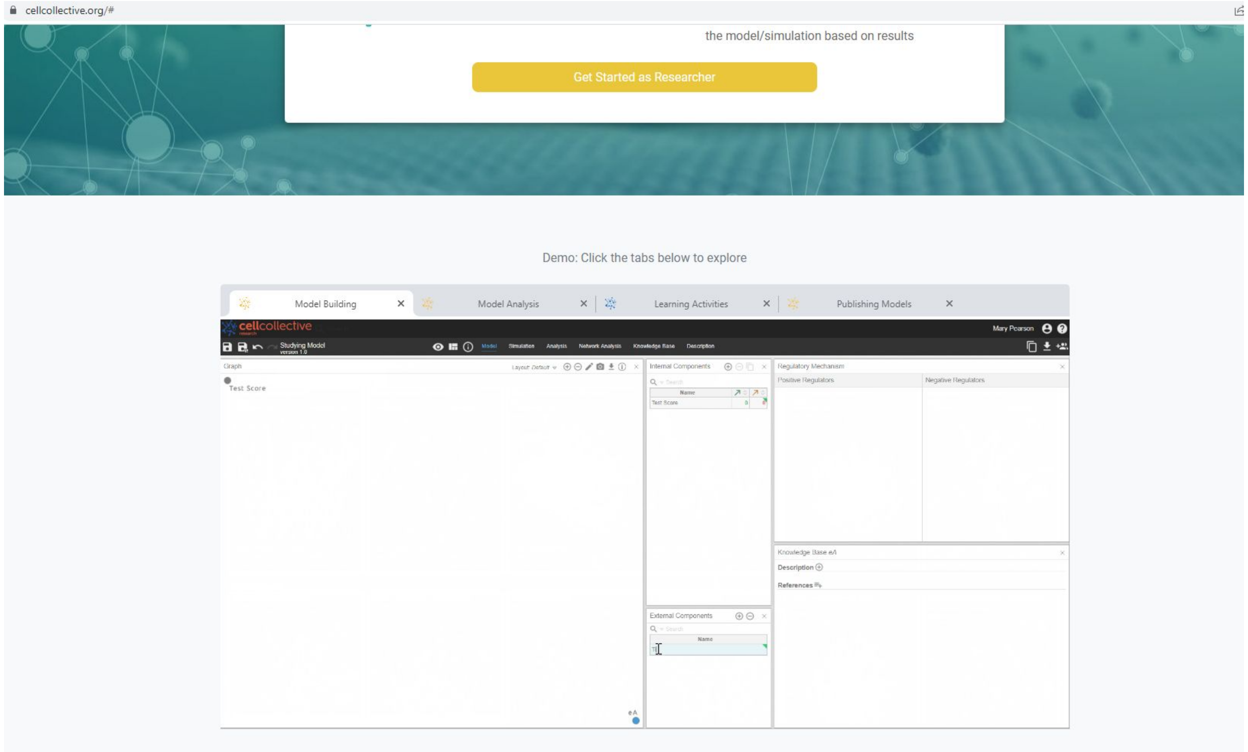

Screenshot of the interactive demo to build and analyze logical models in Cell Collective. https://cellcollective.org/#

B) The link to the YouTube Tutorial of Cell Collective

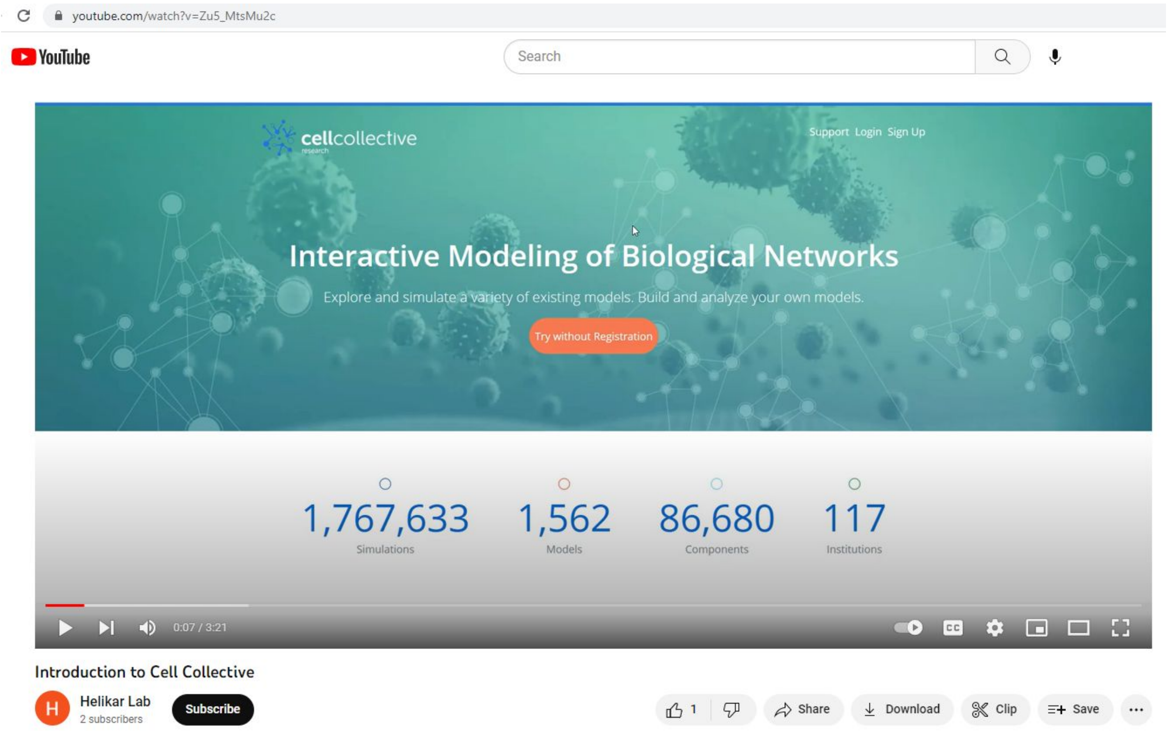

Screenshot of the introduction to Cell Collective in YouTube.
https://www.youtube.com/watch?v=Zu5_MtsMu2c

C1) Example of the Cell Collective environment- Model panel

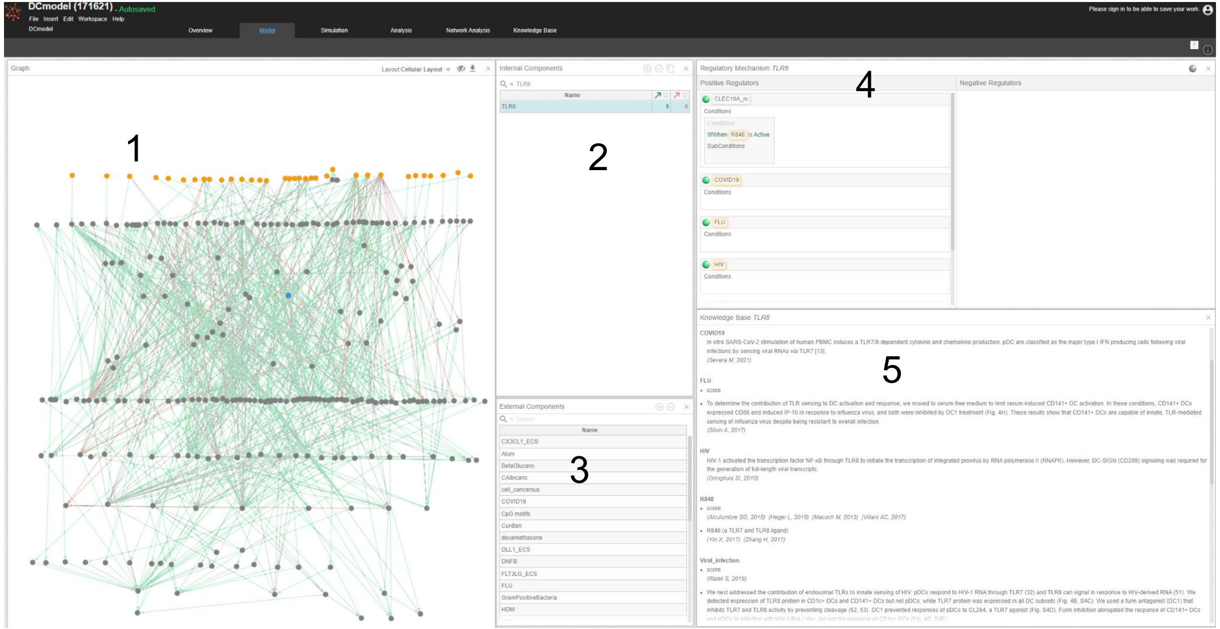

1-Overview of the DC model with nodes and interactions.

2-Internal components (green indicated the number of positive regulators and red, the number of negative regulators)

3-List of external components (search bar for quick screen)

4-Regulatory mechanism: example of TLR8 (green indicated the positive regulators)

5-Knowledge base: literature related to TLR8

C2) Example of the Cell Collective environment- Simulation panel

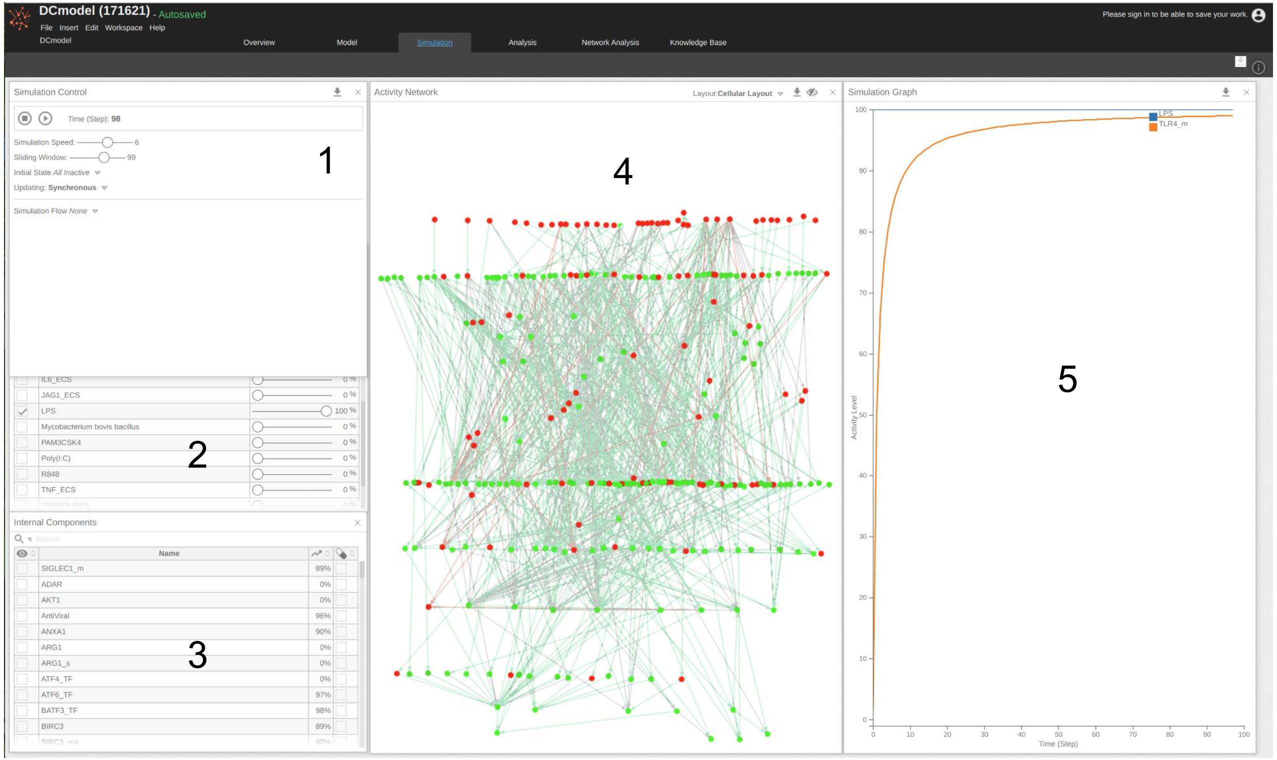

1-To simulate DC model: the panel includes speed, steps, stop and start buttons.

2-To chose the external component and percentage of activity as inputs : example of LPS, with 100% activity.

3-To chose internal components to visualize on the graph (oeil button), to activate or inhibit (pill button). Example provided is TLR4, receptor of LPS.

4-Activity network in real time showing active and inactive nodes. The green indicates active and red, inactive ones.

5-Simulation graph for visualization of the simulation: example of TLR4 active under LPS environment, legends provided on the top right corner.

C3) Example of the Cell Collective environment- Analysis panel

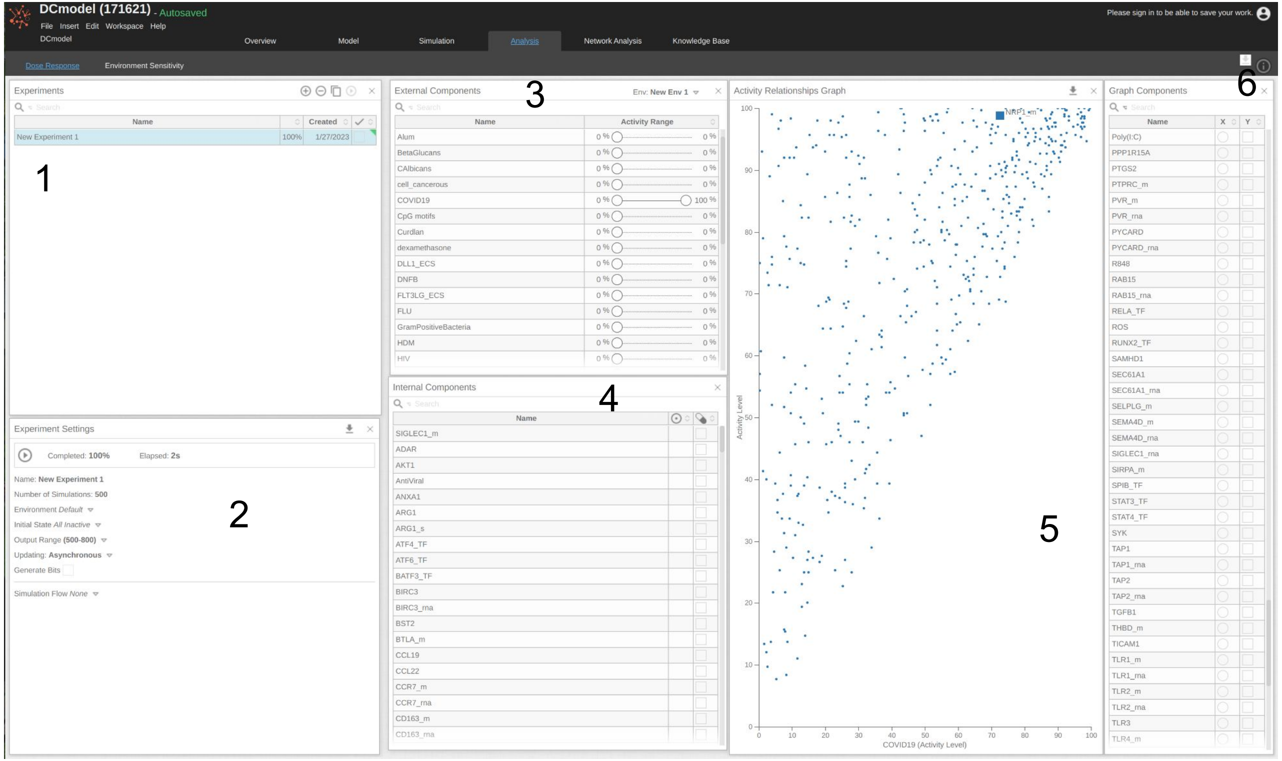

1-To create a file for new experiment

2-To chose experimental settings: name of the file, number of simulation, environment, initial state, range of simulation. Data can be downloaded using the arrow button.

3-To choose external component and the desired activity level: example of Covid activity range 0-100% while all others are at 0.

4-To choose one or multiple internal components to activate or inhibit using the pill button.

5-Activity relationship Graph for visualization: example of NRP1 receptor for Covid.

6-In the graph components, choose what to display in the graph for x and y axis

C4) Example of the Cell Collective environment- Knowledge Base

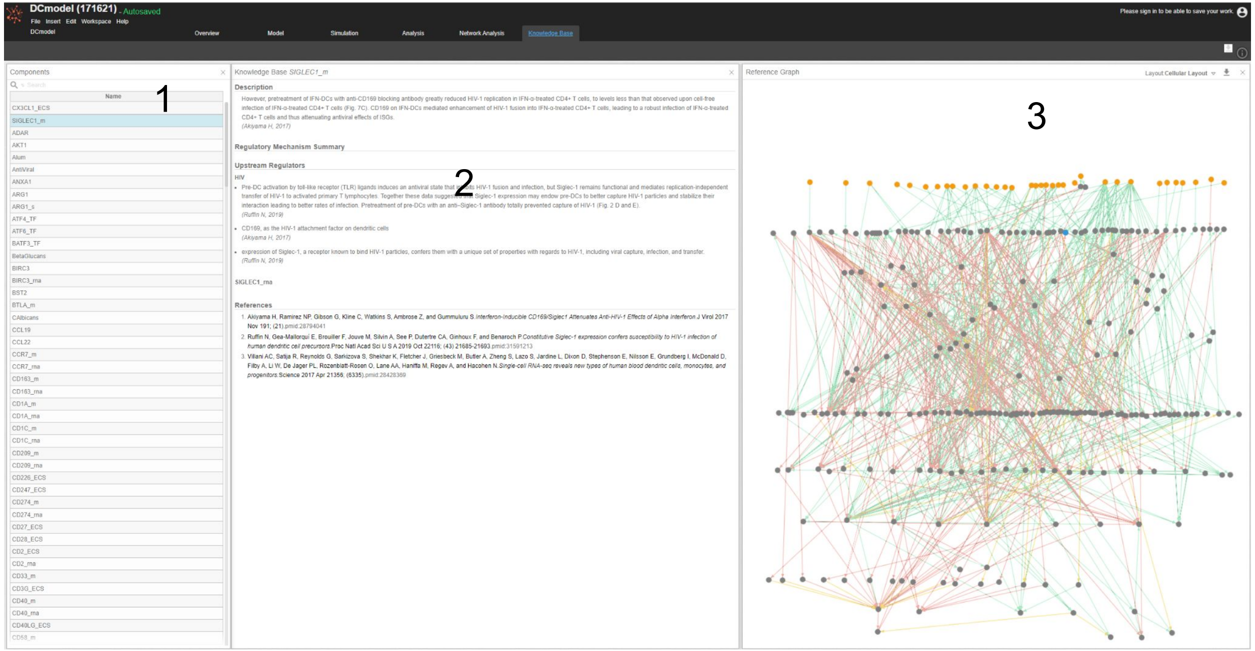

1-List of the internal and external components of the model

2-Knowledge base: description, regulators and references are provided (example of Siglec1).

3-Reference Graph: overview of the model

C5) Example of the Cell Collective environment- Download SBML-qual file

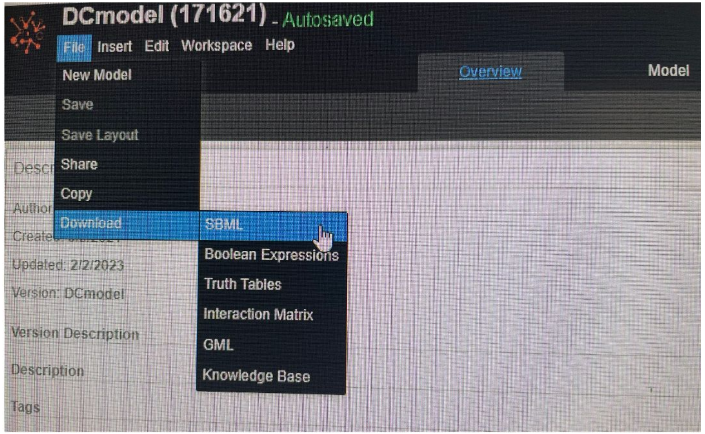

Screenshot of the Cell Collective platform that allows the users to download the DC model's SBML-qual, Boolean expressions, truth tables, interaction matrix, GML, and knowledge base files.
